# Generation mechanism of RANKL^+^ effector memory B cells: relevance to the pathogenesis of rheumatoid arthritis

**DOI:** 10.1186/s13075-016-0957-6

**Published:** 2016-03-16

**Authors:** Yuri Ota, Hiroaki Niiro, Shun-ichiro Ota, Naoko Ueki, Hirofumi Tsuzuki, Tsuyoshi Nakayama, Koji Mishima, Kazuhiko Higashioka, Siamak Jabbarzadeh-Tabrizi, Hiroki Mitoma, Mitsuteru Akahoshi, Yojiro Arinobu, Akiko Kukita, Hisakata Yamada, Hiroshi Tsukamoto, Koichi Akashi

**Affiliations:** Department of Medicine and Biosystemic Science, Graduate School of Medical Sciences, Kyushu University, 3-1-1 Maidashi Higashi-ku, Fukuoka, 812-8582 Japan; Clinical Education Center, Kyushu University Hospital, 3-1-1 Maidashi Higashi-ku, Fukuoka, 812-8582 Japan; Department of Microbiology, Faculty of Medicine, Saga University, 5-1-1 Nabeshima, Saga, 849-8501 Japan; Division of Host Defense, Medical Institute of Bioregulation, Kyushu University, 3-1-1 Maidashi Higashi-ku, Fukuoka, 812-8582 Japan

**Keywords:** B cells, RANKL, Rheumatoid arthritis

## Abstract

**Background:**

The efficacy of B cell-depleting therapies for rheumatoid arthritis underscores antibody-independent functions of effector B cells such as cognate T–B interactions and production of pro-inflammatory cytokines. Receptor activator of nuclear factor κB ligand (RANKL) is a key cytokine involved in bone destruction and is highly expressed in synovial fluid B cells in patients with rheumatoid arthritis. In this study we sought to clarify the generation mechanism of RANKL^+^ effector B cells and their impacts on osteoclast differentiation.

**Methods:**

Peripheral blood and synovial fluid B cells from healthy controls and patients with rheumatoid arthritis were isolated using cell sorter. mRNA expression of RANKL, osteoprotegerin, tumor necrosis factor (TNF)-α, and Blimp-1 was analyzed by quantitative real-time polymerase chain reaction. Levels of RANKL, CD80, CD86, and CXCR3 were analyzed using flow cytometry. Functional analysis of osteoclastogenesis was carried out in the co-culture system using macrophage RAW264 reporter cells.

**Results:**

RANKL expression was accentuated in CD80^+^CD86^+^ B cells, a highly activated B-cell subset more abundantly observed in patients with rheumatoid arthritis. Upon activation via B-cell receptor and CD40, switched-memory B cells predominantly expressed RANKL, which was further augmented by interferon-γ (IFN-γ) but suppressed by interleukin-21.

Strikingly, IFN-γ also enhanced TNF-α expression, while it strongly suppressed osteoprotegerin expression in B cells. IFN-γ increased the generation of CXCR3^+^RANKL^+^ effector B cells, mimicking the synovial B cell phenotype in patients with rheumatoid arthritis. Finally, RANKL^+^ effector B cells in concert with TNF-α facilitated osteoclast differentiation in vitro.

**Conclusions:**

Our current findings have shed light on the generation mechanism of pathogenic RANKL^+^ effector B cells that would be an ideal therapeutic target for rheumatoid arthritis in the future.

**Electronic supplementary material:**

The online version of this article (doi:10.1186/s13075-016-0957-6) contains supplementary material, which is available to authorized users.

## Background

Rheumatoid arthritis (RA) is a prototypical autoimmune disease characterized by joint inflammation and bone destruction. The synovium of RA exhibits abundant accumulation of immune and inflammatory cells such as dendritic cells, T cells, B cells, macrophages and neutrophils [1]. The emergence of autoantibodies such as anti-citrullinated protein antibodies (ACPA) and rheumatoid factors (RF) in the preclinical stage of RA underscores an autoimmune-driven process in this disease [2].

Among current biological therapies targeting immune cells in RA, the advent of B cell-depleting agents, such as rituximab (RTX), has prompted reappraisal of the role of B cells in the pathogenesis of this disease [3–5]. Most intriguingly, RTX significantly inhibits synovial inflammation and bone destruction without reducing the titers of autoantibodies. Pathogenic B cells in RA are thus most likely to function as potent effectors mainly in an antibody-independent manner, presumably via cognate T–B interactions and release of pro-inflammatory cytokines [6–9].

Receptor activator of nuclear factor κB ligand (RANKL) is a pro-inflammatory cytokine of the tumor necrosis factor (TNF) family and is a key positive regulator of osteoclast development and activation that is pertinent to bone destruction. Previous studies showed that RANKL is highly expressed in synovial fibroblasts and activated T cells in RA [10–12]. A recent study, however, suggests that synovial B cells are another important source of RANKL in RA [13]. In addition, Fc-receptor-like 4 (FcRL4)^+^ B cells, a unique B-cell subset observed in RA synovium but not in the peripheral blood (PB), are proposed as potent effectors expressing RANKL at high levels, although the impact of RANKL^+^ B cells on osteoclastogenesis has yet to be clarified [14].

The following issues remain to be addressed. First, it remains largely elusive how RANKL^+^ effector B cells are generated in RA. Given that T cell-derived cytokines play a crucial role in T–B collaboration in RA, whether these cytokines affect the generation of RANKL^+^ effector B cells is of potential interest. In addition, since B cells are also a potential source of negative regulators for osteoclastogenesis, such as osteoprotegerin (OPG), a decoy receptor for RANKL, the impact of RANKL^+^ effector B cells on osteoclastogenesis needs to be carefully evaluated [15–18].

In this study, based on the key finding that RANKL was expressed at high levels in CD80^+^CD86^+^ B cells from patients with RA, we demonstrate that, upon activation via B-cell receptor (BCR) and CD40, human switched-memory B cells predominantly expressed RANKL, which was further augmented by interferon (IFN)-γ but suppressed by interleukin (IL)-21. Notably, IFN-γ also enhanced TNF-α expression, while it strongly suppressed OPG expression in B cells. IFN-γ increased expression of CXC motif chemokine receptor 3 (CXCR3) in RANKL^+^ B cells, mimicking the synovial B cell phenotype in RA. Finally, RANKL^+^ effector B cells in concert with TNF-α facilitated osteoclast differentiation in vitro. Together, these findings have thrown light on the generation mechanism of RANKL^+^ effector memory B cells that would be an ideal therapeutic target for RA in the future.

## Methods

### Patients and controls

Patients with RA met with the 1987 American College of Rheumatology classification criteria. PB was obtained from 24 patients with RA (PBRA; 3 males and 21 females, 21 to 75 years old, average age 60.0 years). Synovial fluid (SF) was obtained from 8 patients with RA (SFRA; 1 male and 7 females, 22 to 85 years old, average age 60.6 years). PB from healthy controls (PBHC) matched to the RA patients’ gender and age served as controls. Patient details are provided in Additional file 1 (Table S1). Informed consent was obtained from all subjects in accordance with the Declaration of Helsinki. The Institutional Review Board of Kyushu University Hospital approved all research on human subjects.

### Reagents

An affiniPure F (ab’) Fragment Goat Anti-Human IgA/IgG/IgM (H + L) (BCR, 10 μg/ml) was purchased from Jackson ImmunoResearch (West Grove, PA, USA). Anti-human CD40 monoclonal antibody (CD40, 2 μg/ml), recombinant human cytokines (TNF-α (100 ng/ml), IFN-γ (20 ng/ml), IL-4 (20 ng/ml), IL-17 (100 ng/ml), IL-2 (100 ng/ml), IL-6 (100 ng/ml)), recombinant human IL-6 Receptor (100 ng/ml) and OPG (100 ng/ml)) were from R&D Systems (Minneapolis, MN, USA). Recombinant human CXC motif chemokine ligand 10 (CXCL10, 100 ng/ml) was from PeproTech Inc (Rocky Hill, NJ, USA). Recombinant human IL-21(20 ng/ml) was from Miltenyi Biotec (Auburn, CA, USA). CpG ODN 2006, type C (Toll-like receptor 9 (TLR9), 0.1 μM) was from Gene Design Inc (Osaka, Japan). A fully human monoclonal antibody against RANKL (αRANKL, 100 ng/ml) was from Amgen Inc (Thousand Oaks, CA, USA).

### Isolation and cell sorting of B cell subsets

Mononuclear cells were isolated from PB and SF using a density centrifugation with LSM (MP Biomedicals, LLC, Santa Ana, CA, USA). B cells were isolated by positive selection with Dynabeads M450 CD19 and DETACHaBEAD CD19 (Invitrogen, Carlsbad, CA, USA) as previously described [19]. As we previously showed, only negligible levels of artificial activation of B cells occurred immediately after positive selection [20]. To avoid unwanted further stimulation, cells were normally rested on ice prior to any stimulation. Isolated B cells exhibited greater than 99.5 % viability and more than 95 % purity, confirmed by flow cytometry. Cells were stained with mouse or rabbit monoclonal antibody (mAb) against human CD19, IgD, CD27, CD80, CD86, CD183 (CXCR3), CD254 (RANKL) and CD307d (FcRL4) (all from BioLegend, San Diego, CA, USA). Naïve (IgD^+^CD27^–^), IgD^+^-memory (IgD^+^CD27^+^), double-negative (IgD^–^CD27^–^) and switched-memory (IgD^–^CD27^+^) B cell subsets were purified by flow cytometry.

### Quantitative real-time polymerase chain reaction

Total RNA was extracted from primary B cells using Isogen II reagent (Nippon Gene, Tokyo, Japan). First-strand complementary DNA (cDNA) was synthesized using a SuperScript III First-Strand Synthesis SuperMix (Invitrogen). Quantitative real-time polymerase chain reaction (PCR) was performed in the ABI Prism 7500 Sequence Detector (Applied Biosystems, Foster City, CA, USA). The reactions were performed in triplicate wells in 96-well plates. TaqMan target mixes for TNFSF11 (Hs00243533_m1), TNF (Hs00174128_m1), TNFRSF11B (Hs00900358_m1), PRDM1 (Hs00153357_m1) and CXCR3 (Hs00171041_m1) were all purchased from Applied Biosystems. 18S ribosomal RNA was separately amplified in the same plate as an internal control for variation in the amount of cDNA in PCR. The collected data were analyzed using Sequence Detector software (Applied Biosystems). Data were expressed as the fold-change in gene expression relative to the expression in control cells.

### Plasmid construction

The pE2-ctsk-Venus plasmid expressing Venus protein under the control of cathepsin K (ctsk) promoter was constructed. To prepare this plasmid, the CMV promoter of pE2-Crimson-N1 (Clontech laboratories Inc, CA, USA) was first replaced by the mouse Ctsk promoter fragment (–1676 to –48) [21]. The fragment including Crimson coding region of the resulting plasmid was then replaced by the fragment including Venus coding region derived from pCS2-Venus vector kindly provided by Dr. A. Miyawaki (Brain Science Institute, RIKEN, Japan).

### Generation of RAW264 Venus reporter cells

The pE2-ctsk-Venus plasmid was linearized by ApaLI digestion and transfected into RAW-D cells (sub-clone of RAW264 [22]) by electroporation at 300 V and 950 μF using a Gene Pulser Xcell (BioRad). Twenty-four hours after transfection, cells were then selected by G418 (500 μg/ml) for 3 weeks to establish RAW-Ctsk-Venus cell lines that can express Venus protein by addition of RANKL (30 ng/ml). Among several clones, RAW-ctsk-Venus1 (RAW-Venus1) clone with highest induction of Venus protein was selected.

### Analysis of osteoclast differentiation (osteoclastogenesis)

We first confirmed that differentiation of RAW-Venus1 cells into Venus-positive pre-osteoclasts and osteoclasts were induced by RANKL in a dose-dependent manner (Additional file 2: Figure S1). TNF-α exerted synergistic effects on RANKL-induced osteoclast differentiation. In addition, both anti-RANKL Ab and OPG strongly reduced the number of Venus-positive cells, indicating the inhibition of osteoclast differentiation. RAW-Venus1 cells were cultured at 2.25 × 10^4^ cells/ml for 1 day in a 96-well plate and served as osteoclast precursors. These cells were co-cultured with B cells at 2.25 × 10^4^ cells/well for 2 days and Venus-positive cells were counted by Electrophysiological Microscopes (Keyence, Osaka, Japan).

### Statistical analysis

Numerical data in the in vitro experiments were presented as mean of the different experiments and standard error of the mean (SEM). The significance of the differences was determined by Student's *t*-test for comparing differences between two groups, and one-way analysis of variance for comparing differences between multiple groups. In some experiments, Dunnett’s test was applied. Numerical data in patient-sample analyses were presented as mean, and the significance of differences (SD) was determined by Student’s *t*-test or non-parametric Mann-Whitney U-test according to distributions. For all tests, *P* values less than 0.05 were considered significant. Statistical analysis was performed with GraphPad Prism 6 (GraphPad Software, La Jolla, CA, USA).

## Results

### Activation via BCR and CD40 induces RANKL expression in CD80^+^CD86^+^ B cells

**Fig. 1:**
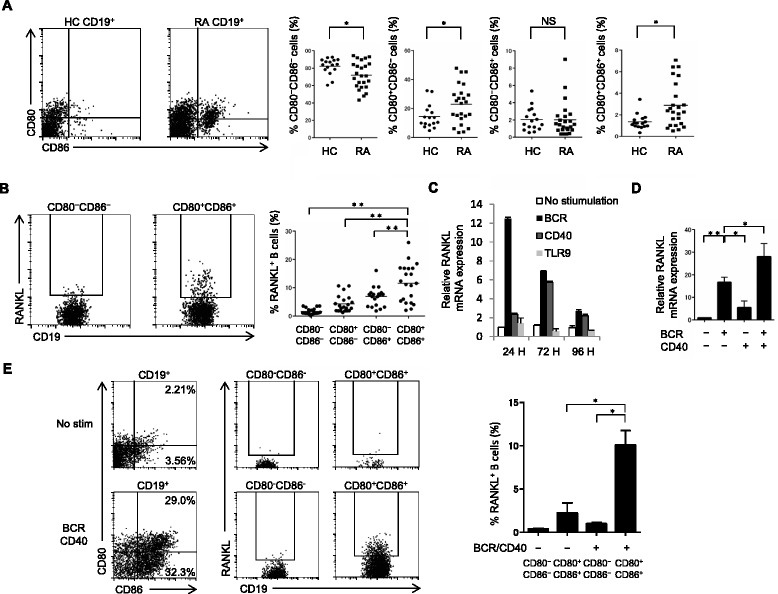
Activation via B-cell receptor (*BCR*) and CD40 induces receptor activator of nuclear factor kappa-B ligand (*RANKL*) expression in CD80^+^CD86^+^ B cells. **a** Freshly isolated PB B cells were stained with CD80 and CD86, and the proportion of each subset from 16 healthy controls (*HC*) and 24 patients with rheumatoid arthritis (*RA*) was analyzed by flow cytometry. **b** RANKL expression was analyzed in each subset of 24 patients with RA. Representative data are shown in (**a**) and (**b**). *Horizontal lines* show the mean (*P < 0.05, **P < 0.005). **c** Purified B cells from HC were stimulated for 24, 72 and 96 hours via BCR, CD40 or Toll-like receptor 9 (*TLR9*). **d** Purified B cells from HC were stimulated for 24 hours via BCR and/or CD40. RANKL mRNA expression was analyzed by quantitative PCR in (**c**) and (**d**). The values are the mean ± SEM of three independent experiments (n = 5, *P < 0.05, **P < 0.005). **e** Purified B cells from HC were incubated for 48 hours with or without BCR/CD40 stimulation, and CD80, CD86 and RANKL expression were analyzed by flow cytometry. The results shown are representative of three independent experiments. The values are the mean ± SEM (n = 4, *P < 0.05). *No stim* no stimulation, *NS* not significant

Although a previous study showed that RANKL^+^ B cells are barely detected in human PB [13], we hypothesized that a specific B-cell subpopulation might express high levels of RANKL. CD80 and CD86 are surface markers representing the status of highly activated B cells that make cognate interaction with activated T cells. We first tested the abundance of B-cell subsets defined by CD80 and CD86 staining in healthy controls (HC) and patients with RA. The proportion of CD80^+^CD86^+^ B cells was significantly higher in patients with RA than in HC (Fig. 1a). In addition, such highly activated (CD80^+^CD86^+^) B cells significantly expressed RANKL at higher levels than non-activated (CD80^–^CD86^–^) B cells (Fig. 1b) in patients with RA, suggesting that robust B-cell activation is required for RANKL expression.

We thus sought to determine what conditions could induce RANKL expression in B cells from HC. Robust activation of B cells via BCR, CD40 or TLR9 is involved in the pathogenesis of autoimmune diseases. Compared with TLR9, stimulation of BCR and, to a lesser extent, CD40 significantly induced RANKL expression in B cells (Fig. 1c). Co-stimulation of BCR and CD40 further enhanced RANKL expression (Fig. 1d) and generated CD80^+^CD86^+^ B cells expressing RANKL at high levels (Fig. 1e). These suggest that robust activation of B cells via BCR and CD40 induces RANKL expression in CD80^+^CD86^+^ B cells.

### BCR/CD40-induced RANKL expression in switched-memory B cells is augmented by IFN-γ but suppressed by IL-21

**Fig. 2:**
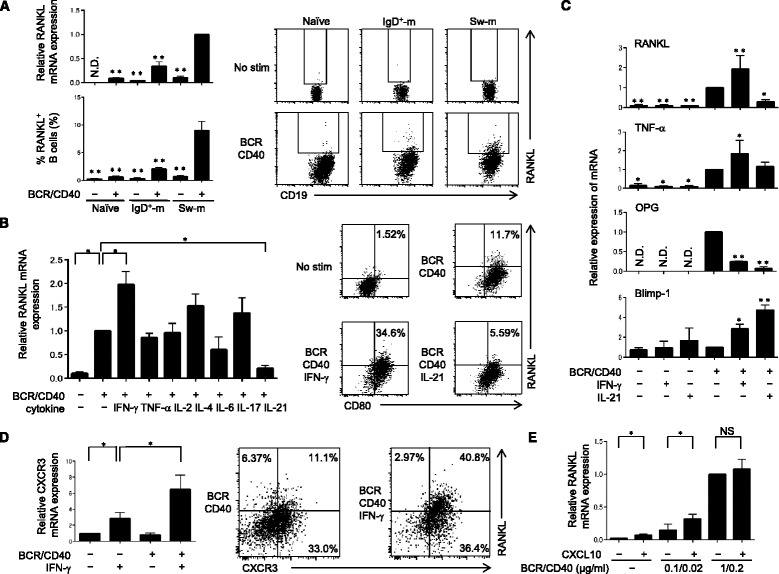
B-cell receptor (*BCR*)/CD40-induced receptor activator of nuclear factor kappa-B ligand (*RANKL*) expression in switched-memory B cells is augmented by interferon gamma (*IFN-γ*) but is suppressed by interleukin-21 (*IL-21*). **a** Naïve (IgD^+^CD27^–^), IgD^+^-memory (*IgD* ^*+*^ *-m*, IgD^+^CD27^+^) and switched-memory (*Sw-m*, IgD^–^CD27^+^) B cell subsets from HC were purified by flow cytometry and stimulated with or without BCR/CD40. RANKL expression at mRNA and protein levels was detected by quantitative PCR and flow cytometry, respectively. The values are the mean ± SEM of three independent experiments (n = 5, **P < 0.005, with reference to BCR/CD40-stimulated sw-m B cells). **b** Purified switched-memory B cells from HC were incubated with IFN-γ, tumor necrosis factor alpha (*TNF-α*), IL-2, IL-4, IL-6 + IL-6 receptor, IL-17, or IL-21 in addition to BCR/CD40 simulation. RANKL mRNA expression was analyzed by quantitative PCR (n = 4, *P < 0.05, with reference to BCR/CD40-stimulated sw-m B cells). Expression of RANKL and CD80 was detected by flow cytometry (n = 4). **c** Purified switched-memory B cells from HC were stimulated via BCR/CD40, IFN-γ or IL-21, and mRNA expression of indicated molecules was analyzed by quantitative PCR. The values are the mean ± SEM of three independent experiments (n = 4, *P < 0.05, **P < 0.005, with reference to BCR/CD40-stimulated sw-m B cells). **d** Purified switched-memory B cells from HC were cultured with BCR/CD40 stimulation and/or IFN-γ, and CXCR3 mRNA expression was analyzed (n = 3, *P < 0.05, with reference to IFN-γ-stimulated sw-m B cells). Expression of RANKL and CXCR3 was detected by flow cytometry (n = 7). **e** Purified switched-memory B cells from HC were stimulated with the indicated dose of BCR/CD40 in the presence or absence of CXCL10 (100 ng/ml) and RANKL mRNA expression was analyzed. BCR/CD40 stimulation was carried out at suboptimal or optimal dose (n = 3, *P < 0.05). **a**–**e** mRNA expression of each molecule was detected by quantitative PCR after 24 hours culture. Expression of RANKL, CD80 and CXCR3 was detected after 48 hours culture. *OPG* osteoprotegerin, *N.D.* not determined, *No stim* no stimulation, *NS* not significant

To further determine the differences of RANKL expression in B-cell subsets, we sorted naïve B cells, IgD^+^-memory B cells and switched-memory B cells from HC. Without stimulation, RANKL was only weakly expressed in all subsets; however, BCR/CD40 stimulation induced expression of RANKL mRNA and protein at high levels predominantly in switched-memory B cells (Fig. 2a).

Co-stimulation of BCR and CD40 in B cells mimics T cell-dependent responses in vivo. Given that activated T cells produce various cytokines, we next questioned whether such cytokines could modulate BCR/CD40-induced RANKL expression in switched-memory B cells. Among cytokines tested, IFN-γ remarkably augmented RANKL expression, while IL-21 significantly suppressed it at both mRNA and protein levels (Fig. 2b, c). Of note, BCR/CD40 stimulation of switched-memory B cells augmented CD80 expression, which was however not affected by either IFN-γ or IL-21 (Fig. 2b, right panel). These results suggest that RANKL expression is regulated by a mechanism distinct from CD80 expression in B cells with IFN-γ and IL-21.

B cells can also produce important regulators other than RANKL for osteoclastogenesis. The pro-inflammatory cytokine TNF-α concerts with RANKL to induce osteoclastogenesis, while OPG is decoy receptor for RANKL and functions as a negative regulator of osteoclastogenesis. Notably, IFN-γ stimulation increased TNF-α expression, while it strongly suppressed OPG expression in switched-memory B cells (Fig. 2c), thus tipping the balance more towards osteoclastogenesis. IL-21 stimulation suppressed both RANKL and OPG expression, while it upregulated expression of Blimp-1, suggesting that IL-21 plays a critical role in plasma cell differentiation compared to osteoclastogenesis [23]. Together, these results suggest that T cell-derived IFN-γ strongly augments the generation of RANKL^+^ effector memory B cells.

### IFN-γ increases CXCR3^+^RANKL^+^ effector memory B cells

The chemokine CXCL10, a ligand for CXCR3, is increased in SFRA and attracts leukocytes to the inflammatory lesion [24–27]. Although BCR/CD40 stimulation alone was without effect on CXCR3 expression, IFN-γ significantly induced CXCR3 expression that was further augmented by BCR/CD40 stimulation in switched-memory B cells (Fig. 2d). Notably, CXCR3^+^ switched-memory B cells mainly expressed RANKL that was further pronounced by IFN-γ (Fig. 2d, right panel).

A previous report showed that CXCL10 stimulation induces RANKL expression in CD4^+^ T cells in RA [28]. We thus tested the effect of CXCL10 on RANKL expression in switched-memory B cells. CXCL10 stimulation increased RANKL expression with or without suboptimal BCR/CD40 stimulation (Fig. 2e). These results suggest that IFN-γ increases the generation of CXCR3^+^RANKL^+^ effector memory B cells, which are in turn recruited to the inflammatory lesions such as the synovium of RA patients. Thus, CXCL10 released at the inflammatory sites not only attracts CXCR3^+^ switched-memory B cells but also enhances their potential to produce RANKL.

### CXCR3^+^RANKL^+^ B cells accumulate in SF of patients with RA

**Fig. 3:**
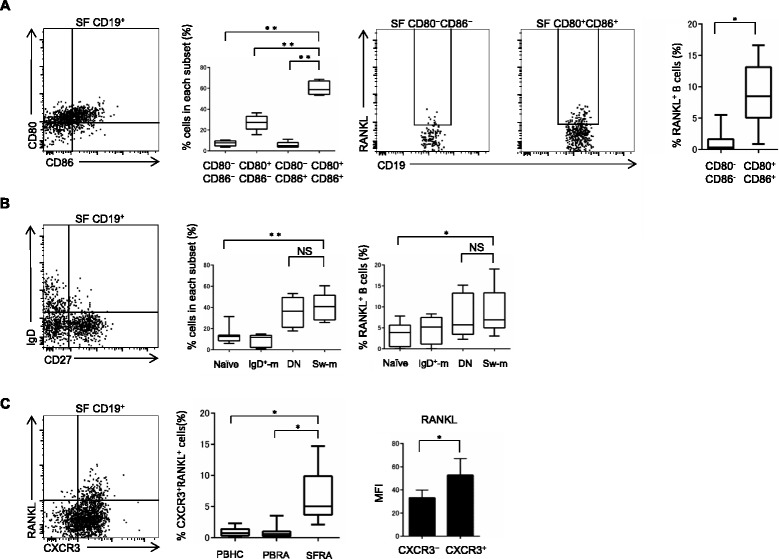
CXCR3^+^RANKL^+^ B cells accumulate in synovial fluid (*SF*) of patients with rheumatoid arthritis (*SFRA*). **a** CD80 and CD86 expression was analyzed in SF B cells from RA patients (n = 8). Receptor activator of nuclear factor kappa-B ligand (*RANKL*) expression in CD80^–^CD86^–^ and CD80^+^CD86^+^ SF B cells was analyzed by flow cytometry. **b** The proportion of naïve (IgD^+^CD27^–^), IgD^+^-memory (*IgD* ^*+*^ *-m*, IgD^+^CD27^+^), double-negative (*DN*) and switched-memory (*Sw-m*, IgD^–^CD27^+^) B cells in SF, and RANKL expression of each subset was analyzed (n = 8). **c** The proportion of CXCR3^+^RANKL^+^ B cells was analyzed in peripheral blood of healthy controls (*PBHC*, n = 13), peripheral blood of patients with RA (*PBRA*, n = 18) and SFRA (n = 8). CXCR3^+^ and CXCR3^–^ SF B cells from patients with RA were analyzed for mean fluorescence intensity (*MFI*) of RANKL expression by flow cytometry (n = 8, *P < 0.05). **a**–**c** Box plots represent the median values, quartiles and extremes (*P < 0.05, **P < 0.005). Representative data are shown. *NS* not significant

We next questioned whether SF B cells in patients with RA exhibit similar phenotype to CXCR3^+^RANKL^+^ effector memory B cells generated in vitro. The proportion of CD80^+^CD86^+^ B cells was significantly higher in SF (Fig. 3a) than in PB (Fig. 1a). Consistent with Fig. 1b, highly activated (CD80^+^CD86^+^) B cells expressed RANKL at high levels (Fig. 3a, far right panel). In addition, double-negative (IgD^–^CD27^–^, a subset also including abundant effector memory cells), and switched-memory (IgD^+^CD27^+^) B cells were predominantly enriched in SFRA (Fig. 3b, left panel), and RANKL expression in both subsets was significantly higher than that in naïve B cells (Fig. 3b, far right panel). Furthermore, the proportion of CXCR3^+^RANKL^+^ B cells was significantly higher in SFRA than in PBHC and PBRA (Fig. 3c, middle panel). Consistent with Fig. 2d, synovial CXCR3^+^ B cells expressed RANKL at higher levels than CXCR3^–^ B cells (Fig. 3c, right panel).

Together, these results suggest that the phenotype of RANKL^+^ B cells in SF of patients with RA is quite akin to that of in vitro-generated CXCR3^+^ effector memory B cells, as shown above.

### RANKL^+^ effector memory B cells in concert with TNF-α facilitate osteoclastogenesis

Since switched-memory B cells abundantly produce both positive (RANKL and TNF-α) and negative (OPG) regulators of osteoclastogenesis (Fig. 2c), we directly tested whether switched-memory B cells could induce osteoclast differentiation, using a clone of macrophage RAW264 reporter cells (RAW-Venus1). This in vitro system allowed us to monitor the initiation of osteoclast differentiation by the detection of Venus positive cells.

**Fig. 4:**
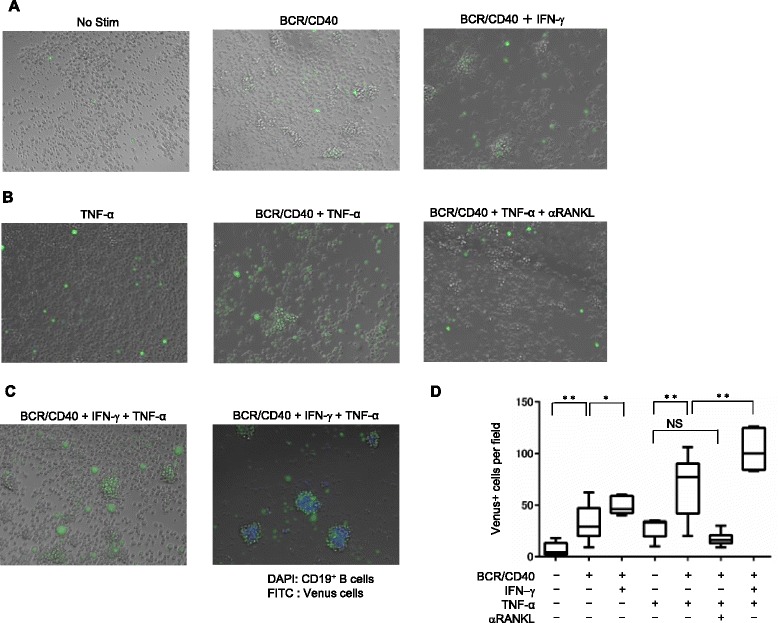
RANKL^+^ effector memory B cells in concert with tumor necrosis factor alpha (*TNF-α*) facilitate osteoclastogenesis. **a**–**c** RAW-Venus1 cells were co-cultured with switched-memory B cells at 2.25 × 10^4^ cells/well for 2 days with or without BCR/CD40 stimulation, interferon gamma (*IFN-γ*, 20 ng/ml), TNF-α (100 ng/ml) or monoclonal antibody against receptor activator of nuclear factor kappa-B ligand (*αRANKL*, 100 ng/ml). Venus positive cells were detected using the fluorescein isothiocyanate (*FITC*) channel. A field equaled an area of 0.39 mm^2^ (magnification × 20). **c** Venus positive cells co-localized with switched-memory CD19^+^ B cells from HC. **d** Venus positive cells were counted in three randomly selected fields of three to five independent experiments. Box plots represent the median values, quartiles and extremes (n = 9, *P < 0.05, **P < 0.005). *BCR* B-cell receptor, *DAPI* 4’6-diamidino-2-phenylindole, *No stim* No stimulation, *NS* not significant

Without stimulation, switched-memory B cells marginally induced Venus positive cells; however, upon BCR/CD40 stimulation they remarkably induced Venus expression, which was further enhanced by the addition of IFN-γ (Fig. 4a, d). In this system, TNF-α alone also induces Venus positive cells and synergistically enhances a RANKL-dependent osteoclast differentiation. In the presence of an excess amount of TNF-α, BCR/CD40-stimulated switched-memory B cells induced more Venus positive cells, a process suppressed by addition of anti-RANKL mAb, validating the involvement of RANKL in this process (Fig. 4b, d). Strikingly, co-stimulation of activated B cells with IFN-γ and TNF-α further increased Venus positive cells, and co-localization of B cells and Venus cells was observed (Fig. 4c, d). Together, these results suggest that, despite their potential to produce OPG, switch-memory B cells induce RANKL^+^ effector cells upon stimulation of BCR/CD40 and IFN-γ, and in concert with TNF-α facilitate osteoclastogenesis.

## Discussion

In this study we demonstrate that RANKL^+^ B cells were enriched in the CD80^+^CD86^+^ subpopulation more frequently in patients with RA. Activation via BCR and CD40 induced switched-memory B cells to express RANKL, which was further augmented by IFN-γ but suppressed by IL-21. IFN-γ also enhanced TNF-α expression, while it strongly suppressed OPG expression in B cells. IFN-γ increased CXCR3^+^RANKL^+^ B cells, mimicking the synovial B-cell phenotype in patients with RA. RANKL^+^ effector memory B cells in concert with TNF-α facilitated osteoclast differentiation in vitro. These findings suggest that CXCR3^+^ RANKL^+^ switched-memory B cells are potent pathogenic effectors in RA.

CD4^+^ T-cell subsets are defined on different patterns of cytokine production that in turn affect B-cell functions. Among T cell-derived cytokines tested, the Th1 cytokine IFN-γ most efficiently upregulated RANKL expression in BCR/CD40-stimulated switched-memory B cells (Fig. 2). Most strikingly, IFN-γ simultaneously inhibited OPG expression, thereby tipping the balance more towards osteoclastogenesis. Indeed, a recent report showed that citrulline-reactive memory Th1 but not Th17 cells are more abundant in the PB of patients with RA [29]. This suggests that RANKL^+^ switched-memory B cells require cognate interaction with Th1 cells to make them fully pathogenic effector B cells in RA. How then are pathogenic Th1 and B cells recruited to and maintained in the inflammatory site such as RA synovium? The chemokine CXCL10, a ligand for CXCR3, is abundantly secreted in RA synovium [24–27]. CXCR3 is a phenotypic marker of Th1 cells and we previously showed that Th1 but not Th17 cells predominate in the joints of RA patients [30]. Likewise, RANKL^+^ B cells in SFRA predominantly expressed CXCR3 (Fig. 3). In addition, we found that CXCL10 stimulation via CXCR3 enhanced RANKL expression in B cells, thus suggesting a vicious cycle for aggravation of bone destruction in RA. It should be noted that anti-CXCL10 therapy shows efficacy in patients with RA [31].

In addition to B cells, synovial fibroblasts and activated T cells are the source of RANKL in RA synovium [10–12]. In mice, RANKL produced by synovial fibroblasts, but not T cells, plays a pivotal role in bone erosions in inflammatory arthritis [32]. The role of B cell-derived RANKL in the pathogenesis of these rodent models of RA remains to be clarified. However, in rodent models of periodontitis (PD), gingival B cells abundantly express RANKL and B cell depletion significantly protects alveolar bone loss [33, 34]. Given that RA and PD share the similar features of inflammation and bone destruction, and the latter is indeed closely related to the pathogenesis of the former [35], it is of potential interest to elucidate the pathogenic role of human RANKL^+^ B cells in RA.

The role of specific cell types in osteoclastogenesis is often simply extrapolated from their potential to express RANKL. However, many cell types, including B cells, simultaneously produce negative regulators of osteoclastogenesis such as OPG [16, 17]. Thus, the impact of B cells on osteoclastogenesis needs to be carefully evaluated.

During the preparation of this manuscript, Meednu et al very recently showed that combinatorial stimulation with anti-CD40 and phorbol 12-myristate 13-acetate (PMA) induced RANKL expression in memory B cells and promoted osteoclast differentiation in vitro [36]. These impressive findings emphasize a potential role of effector B cells in osteoclastogenesis involved in RA. Here, instead of fixed cells frequently used in the co-culture system [36, 37], we applied the novel system using macrophage RAW264 Venus reporter cells to monitor osteoclastogenesis co-cultured with live B cells, and showed that switched-memory B cells did facilitate osteoclastogenesis (Fig. 4). These findings suggest that RANKL and TNF-α, two positive regulators produced by effector memory B cells, would override the inhibitory effects of OPG, thereby favoring towards osteoclastogenesis in particular under inflammatory conditions such as RA.

**Fig. 5:**
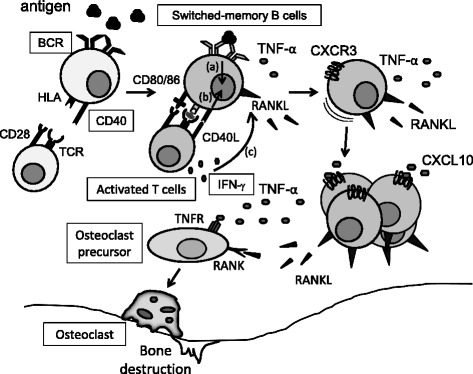
The current hypothetical model in this study. T cell-dependent responses play a pivotal role in B cell-derived receptor activator of nuclear factor kappa-B ligand (*RANKL*) expression in RA. Activation via (**a**) B-cell receptor (*BCR*) and (**b**) CD40 induces switched-memory B cells to express RANKL, a process further enhanced by (**c**) interferon gamma (*IFN-γ*). In this condition B cells express high levels of CXCR3, then facilitating their recruitment into the inflammatory lesions such as the synovium of patients with RA. Notably, CXCL10, a ligand for CXCR3, further enhances the potential of B cells to produce RANKL. Together with their production of tumor necrosis factor alpha (*TNF-α*), RANKL-producing effector memory B cells could thus directly promote osteoclast differentiation and bone destruction. *HLA* human leukocyte antigen, *TCR* T-cell receptor, *RANK* receptor activator of nuclear factor kappa-B, *TNFR* tumor necrosis factor-α receptor

Based on the findings herein, our current model is depicted in Fig. 5. The most puzzling issue is the relationship between RANKL^+^ effector B cells shown in our study and FcRL4^+^ RANKL^+^ effector B cells [14]. We found that FcRL4 expression was detectable in SF B cells of patients with RA; however, it was not appreciably observed in PB B cells of both HC and patients with RA, even upon any stimulation including IFN-γ (data not shown). Notably, although FcRL4^+^ B cells have lower expression of CD21 than FcRL4^–^ B cells [14], RANKL^+^ B cells in our study had higher expression of CD21 than RANKL^–^ B cells (Additional file 3: Figure S2). Whether FcRL4^+^RANKL^+^ B cells originate from CXCR3^+^RANKL^+^ B cells in the PB upon a yet-to-be-identified stimulation, or are novel subsets immigrated from other lymphoid tissues, requires further investigation. Another intriguing issue is whether RANKL^+^ effector B cells have the potential to differentiate into ACPA-producing cells. Given that IL-21, a critical cytokine triggering plasma cell differentiation, inhibited RANKL expression in B cells (Fig. 2), a distinct program might operate in the generation of RANKL^+^ effector B cells. Ongoing experiments are underway to address these outstanding issues.

## Conclusions

This study has uncovered the mechanism by which RANKL^+^ effector B cells are generated in humans. Combined stimulation of BCR and CD40 led to high levels of RANKL expression particularly in switched-memory B cells, which was further augmented by IFN-γ. In addition, IFN-γ facilitated the generation of CXCR3^+^RANKL^+^ memory B cells, reminiscent of synovial B cell phenotype in RA, and these cells in concert with TNF-α induced osteoclast differentiation in vitro. Our current findings would provide a novel clue for the therapeutic strategy to selectively target pathogenic effector B cells in RA, compared with current anti-CD20 therapy in which all B cells are non-selectively depleted.

## Additional files

Additional file 1: Table S1. Clinical characterization of patients with RA included in the study. (DOCX 18 kb) Additional file 2: Figure S1. Analysis of osteoclast differentiation using RAW-Venus1. (A) Differentiation into Venus positive pre-osteoclasts and osteoclasts were induced by RANKL in a dose-dependent manner. TNF-α exerted synergistic effects on RANKL-induced osteoclast differentiation (magnification × 20). (B) Both anti-RANKL Ab and OPG strongly reduced the number of Venus positive cells, indicated the inhibition of osteoclast differentiation (magnification × 20). (C) Venus positive cells were tartrate-resistant acid phosphatase (TRAP)-positive multinucleated cells (magnification × 40). Cells were stained using a commercial TRAP assay (Sigma-Aldrich, St Louis, MO, USA). (A-C) Cells were cultured at 4.5 × 10^4^ cells/ml for 3 days in a 96-well plate. *No stim* No stimulation. (PDF 304 kb) Additional file 3: Figure S2. Phenotypic analysis of RANKL^+^ and RANKL^−^ effector memory B cells. Purified switched-memory B cells from HC were stimulated with BCR/CD40 and IFN-γ for 48 hours. RANKL^+^ and RANKL^−^ cells were analyzed for expression of CD20, CD21, CD95, CD11c, CCR1 and CCR5 using respective Abs (all from BioLegend). Representative data are shown (n = 3–4). (PDF 177 kb)

## Abbreviations

ACPA: Anti-citrullinated protein antibodies
BCR: B-cell receptor
cDNA: Complementary DNA
ctsk: Cathepsin K
CXCL10: CXC motif chemokine ligand 10
CXCR3: CXC motif chemokine receptor 3
FcRL4: Fc-receptor-like 4
HC: Healthy controls
IFN: Interferon
IgD^+^-m: IgD positive un-switched memory
IL: Interleukin
mAb: Monoclonal antibody
OPG: Osteoprotegerin
PB: Peripheral blood
PBHC: Peripheral blood of healthy controls
PBRA: Peripheral blood of patients with rheumatoid arthritis
PCR: Polymerase chain reaction
PD: Periodontitis
RA: Rheumatoid arthritis
RANKL: Receptor activator of nuclear factor kappa-B ligand
RF: Rheumatoid factors
RTX: Rituximab
SD: Significance of differences
SEM: Standard error of the mean
SF: Synovial fluid
SFRA: Synovial fluid of patients with rheumatoid arthritis
TLR9: Toll-like receptor 9
TNF: Tumor necrosis factor

## References

[1] Tak PP, Bresnihan B (2000). The pathogenesis and prevention of joint damage in rheumatoid arthritis: advances from synovial biopsy and tissue analysis. Arthritis Rheum..

[2] Catrina AI, Ytterberg AJ, Reynisdottir G, Malmström V, Klareskog L (2014). Lungs, joints and immunity against citrullinated proteins in rheumatoid arthritis. Nat Rev Rheumatol..

[3] Tak PP, Rigby WF, Rubbert-Roth A, Peterfy CG, van Vollenhoven RF, Stohl W (2011). Inhibition of joint damage and improved clinical outcomes with rituximab plus methotrexate in early active rheumatoid arthritis: the IMAGE trial. Ann Rheum Dis..

[4] Keystone E, Emery P, Peterfy CG, Tak PP, Cohen S, Genovese MC (2009). Rituximab inhibits structural joint damage in patients with rheumatoid arthritis with an inadequate response to tumour necrosis factor inhibitor therapies. Ann Rheum Dis..

[5] Aletaha D, Alasti F, Smolen JS (2013). Rituximab dissociates the tight link between disease activity and joint damage in rheumatoid arthritis patients. Ann Rheum Dis..

[6] Brennan FM, McInnes IB (2008). Evidence that cytokines play a role in rheumatoid arthritis. J Clin Invest..

[7] Braun T, Schett G (2012). Pathways for bone loss in inflammatory disease. Curr Osteoporos Rep..

[8] Braun T, Zwerina J (2011). Positive regulators of osteoclastogenesis and bone resorption in rheumatoid arthritis. Arthritis Res Ther..

[9] Finnegan A, Ashaye S, Hamel KM (2012). B effector cells in rheumatoid arthritis and experimental arthritis. Autoimmunity..

[10] Kotake S, Udagawa N, Hakoda M, Mogi M, Yano K, Tsuda E (2001). Activated human T cells directly induce osteoclastogenesis from human monocytes: possible role of T cells in bone destruction in rheumatoid arthritis patients. Arthritis Rheum..

[11] Komatsu N, Okamoto K, Sawa S, Nakashima T, Oh-hora M, Kodama T (2014). Pathogenic conversion of Foxp3^+^ T cells into T_H_17 cells in autoimmune arthritis. Nat Med..

[12] Takayanagi H, Iizuka H, Juji T, Nakagawa T, Yamamoto A, Miyazaki T (2000). Involvement of receptor activator of nuclear factor kappaB ligand/osteoclast differentiation factor in osteoclastogenesis from synoviocytes in rheumatoid arthritis. Arthritis Rheum..

[13] Yeo L, Toellner KM, Salmon M, Filer A, Buckley CD, Raza K (2011). Cytokine mRNA profiling identifies B cells as a major source of RANKL in rheumatoid arthritis. Ann Rheum Dis..

[14] Yeo L, Lom H, Juarez M, Filer A, Buckley CD, Raza K (2015). Expression of FcRL4 defines a pro-inflammatory, RANKL-producing B cell subset in rheumatoid arthritis. Ann Rheum Dis..

[15] Kong YY, Yoshida H, Sarosi I, Tan HL, Timms E, Capparelli C (1999). OPGL is a key regulator of osteoclastogenesis, lymphocyte development and lymph-node organogenesis. Nature..

[16] Li Y, Toraldo G, Li A, Yang X, Zhang H, Qian WP (2007). B cells and T cells are critical for the preservation of bone homeostasis and attainment of peak bone mass in vivo. Blood..

[17] Yun TJ, Chaudhary PM, Shu GL, Frazer JK, Ewings MK, Schwartz SM (1998). OPG/FDCR-1, a TNF receptor family member, is expressed in lymphoid cells and is up-regulated by ligating CD40. J Immunol..

[18] Fillatreau S, Sweenie CH, McGeachy MJ, Gray D, Anderton SM (2002). B cells regulate autoimmunity by provision of IL-10. Nat Immunol..

[19] Niiro H, Jabbarzadeh-Tabrizi S, Kikushige Y, Shima T, Noda K, Ota S (2012). CIN85 is required for Cbl-mediated regulation of antigen receptor signaling in human B cells. Blood..

[20] Jabbarzadeh-Tabrizi S, Niiro H, Masui M, Yoshimoto G, Iino T, Kikushige Y (2009). T cell leukemia/lymphoma 1 and galectin-1 regulate survival/cell death pathways in human naive and IgM+ memory B cells through altering balances in Bcl-2 family proteins. J Immunol..

[21] Kukita A, Kukita T, Nagata K, Teramachi J, Li YJ, Yoshida H (2011). The transcription factor FBI-1/OCZF/LRF is expressed in osteoclasts and regulates RANKL-induced osteoclast formation in vitro and in vivo. Arthritis Rheum..

[22] Watanabe T, Kukita T, Kukita A, Wada N, Toh K, Nagata K (2004). Direct stimulation of osteoclastogenesis by MIP-1alpha: evidence obtained from studies using RAW264 cell clone highly responsive to RANKL. J Endocrinol..

[23] Berglund LJ, Avery DT, Ma CS, Moens L, Deenick EK, Bustamante J (2013). IL-21 signalling via STAT3 primes human naive B cells to respond to IL-2 to enhance their differentiation into plasmablasts. Blood..

[24] Lee EY, Lee ZH, Song YW (2013). The interaction between CXCL10 and cytokines in chronic inflammatory arthritis. Autoimmun Rev..

[25] Lee EY, Seo M, Juhnn YS, Kim JY, Hong YJ, Lee YJ (2011). Potential role and mechanism of IFN-gamma inducible protein-10 on receptor activator of nuclear factor kappa-B ligand (RANKL) expression in rheumatoid arthritis. Arthritis Res Ther..

[26] Hanaoka R, Kasama T, Muramatsu M, Yajima N, Shiozawa F, Miwa Y (2003). A novel mechanism for the regulation of IFN-gamma inducible protein-10 expression in rheumatoid arthritis. Arthritis Res Ther..

[27] Nanki T, Takada K, Komano Y, Morio T, Kanegane H, Nakajima A (2009). Chemokine receptor expression and functional effects of chemokines on B cells: implication in the pathogenesis of rheumatoid arthritis. Arthritis Res Ther..

[28] Kwak HB, Ha H, Kim HN, Lee JH, Kim HS, Lee S (2008). Reciprocal cross-talk between RANKL and interferon-gamma-inducible protein 10 is responsible for bone-erosive experimental arthritis. Arthritis Rheum..

[29] James EA, Rieck M, Pieper J, Gebe JA, Yue BB, Tatum M (2014). Citrulline-specific Th1 cells are increased in rheumatoid arthritis and their frequency is influenced by disease duration and therapy. Arthritis Rheumatol..

[30] Yamada H, Nakashima Y, Okazaki K, Mawatari T, Fukushi JI, Kaibara N (2008). Th1 but not Th17 cells predominate in the joints of patients with rheumatoid arthritis. Ann Rheum Dis..

[31] Yellin M, Paliienko I, Balanescu A, Ter-Vartanian S, Tseluyko V, Xu LA (2012). A phase II, randomized, double-blind, placebo-controlled study evaluating the efficacy and safety of MDX-1100, a fully human anti-CXCL10 monoclonal antibody, in combination with methotrexate in patients with rheumatoid arthritis. Arthritis Rheum..

[32] Danks L, Komatsu N, Guerrini MM, Sawa S, Armaka M, Kollias G (2015). RANKL expressed on synovial fibroblasts is primarily responsible for bone erosions during joint inflammation. Ann Rheum Dis.

[33] Han X, Kawai T, Eastcott JW, Taubman MA (2006). Bacterial-responsive B lymphocytes induce periodontal bone resorption. J Immunol..

[34] Oliver-Bell J, Butcher JP, Malcolm J, MacLeod MK, Adrados Planell A, Campbell L (2015). Periodontitis in the absence of B cells and specific anti-bacterial antibody. Mol Oral Microbiol..

[35] Hajishengallis G (2015). Periodontitis: from microbial immune subversion to systemic inflammation. Nat Rev Immunol..

[36] Meednu N, Zhang H, Owen T, Sun W, Wang V, Cistrone C (2015). A link between B cells and bone erosion in rheumatoid arthritis: receptor activator of nuclear factor kappa-B ligand production by memory B cells. Arthritis Rheumatol.

[37] Kawai T, Matsuyama T, Hosokawa Y, Makihira S, Seki M, Karimbux NY (2006). B and T lymphocytes are the primary sources of RANKL in the bone resorptive lesion of periodontal disease. Am J Pathol..

