# Supplementary material for: Generation mechanism of RANKL+ effector memory B cells: relevance to the pathogenesis of rheumatoid arthritis
Source: Arthritis Res Ther. 2016 Mar 16;18:67. doi: 10.1186/s13075-016-0957-6 (PMC4793760; doi:10.1186/s13075-016-0957-6)
Supplement: Additional file 1: Table S1. — Clinical characterization of patients with RA included in the study. (DOCX 18 kb) [file 13075_2016_957_MOESM1_ESM.docx]

**Additional file 1: Table S1**

Clinical characterization of patients with RA included in the study

|  | PB B cells  in RA  (n=24) | SF B cells  in RA  (n=8) |
| --- | --- | --- |
| Age, mean (SD), years | 60.0 (12.3) | 60.6 (19.5) |
| Female, n (%) | 21 (87.5) | 7 (87.5) |
| Duration of RA, mean (SD), years | 8.1 (6.4) | 14.9 (9.5) |
| RF-positive, n (%) | 20 (83.3) | 7 (87.5) |
| ACPA-positive, n (%) | 21  (87.5) | 4  (50.0) |
| ESR, mean (SD), mm/h | 25.0 (23.2) | 36.3 (20.4) |
| DAS28 ESR, mean (SD) | 2.8 (1.2) | 3.92 (1.32) |
| Oral steroid use, n (%) | 17 (70.8) | 6 (75.0) |
| Synthetic DMARDs use, n (%) | 20 (83.3) | 7 (87.5) |
| Biologic DMARDs use, n (%) | 8  33.3 | 3  (37.5) |
