# Supplementary material for: Generation mechanism of RANKL+ effector memory B cells: relevance to the pathogenesis of rheumatoid arthritis
Source: Arthritis Res Ther. 2016 Mar 16;18:67. doi: 10.1186/s13075-016-0957-6 (PMC4793760; doi:10.1186/s13075-016-0957-6)

Additional file 2: Figure. S1

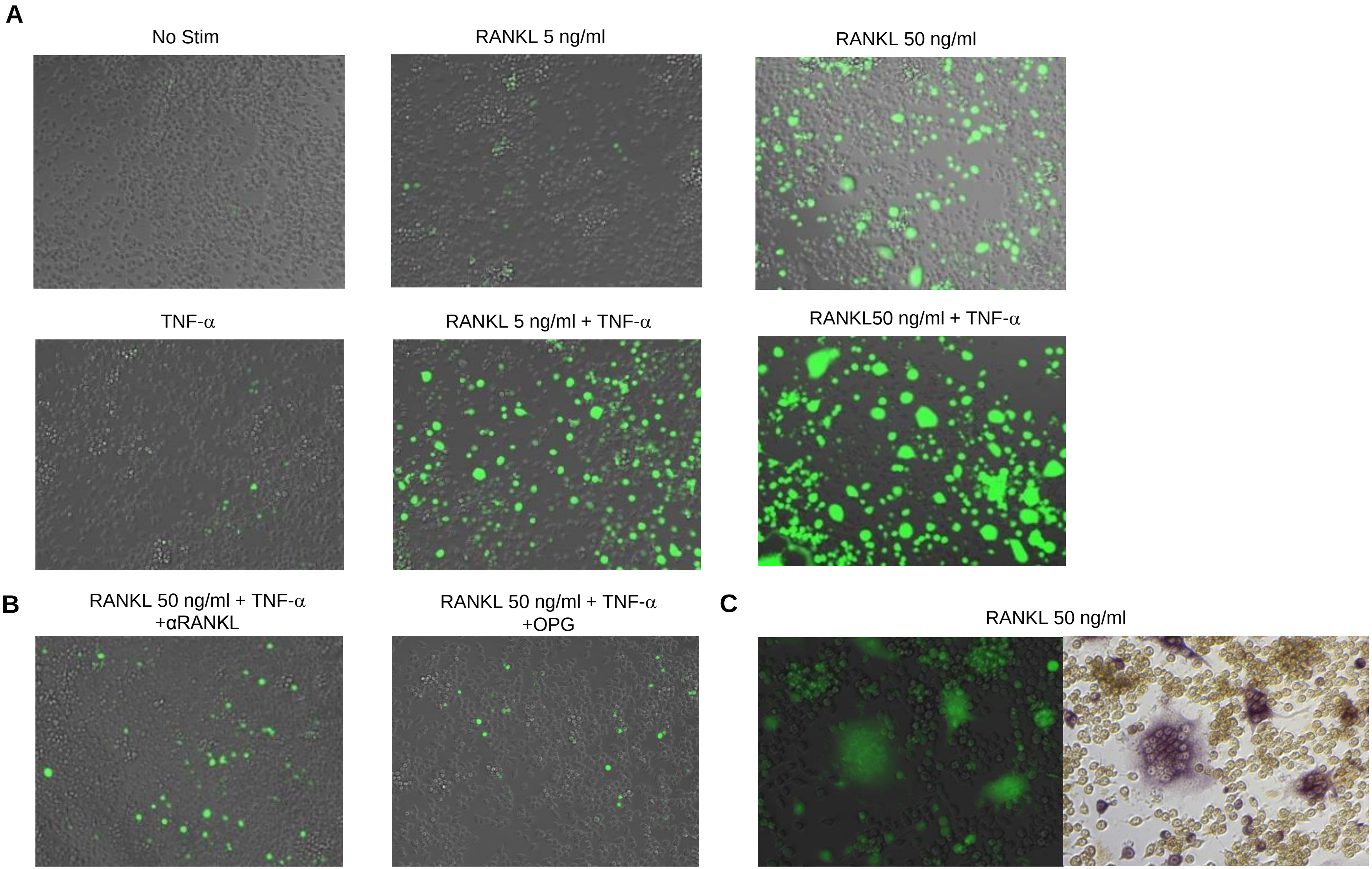

Supplement: Additional file 2: Figure S1. — Analysis of osteoclast differentiation using RAW-Venus1. (A) Differentiation into Venus positive pre-osteoclasts and osteoclasts were induced by RANKL in a dose-dependent manner. TNF-α exerted synergistic effects on RANKL-induced osteoclast differentiation (magnification × 20). (B) Both anti-RANKL Ab and OPG strongly reduced the number of Venus positive cells, indicated the inhibition of osteoclast differentiation (magnification × 20). (C) Venus positive cells were tartrate-resistant acid phosphatase (TRAP)-positive multinucleated cells (magnification × 40). Cells were stained using a commercial TRAP assay (Sigma-Aldrich, St Louis, MO, USA). (A-C) Cells were cultured at 4.5 × 10^4^ cells/ml for 3 days in a 96-well plate. No stim No stimulation. (PDF 304 kb) [file 13075_2016_957_MOESM2_ESM.pdf]
