# Supplementary figures and images for: Generation mechanism of RANKL+ effector memory B cells: relevance to the pathogenesis of rheumatoid arthritis
Source: Arthritis Res Ther. 2016 Mar 16;18:67. doi: 10.1186/s13075-016-0957-6 (PMC4793760; doi:10.1186/s13075-016-0957-6)

Additional file 3: Figure. S2

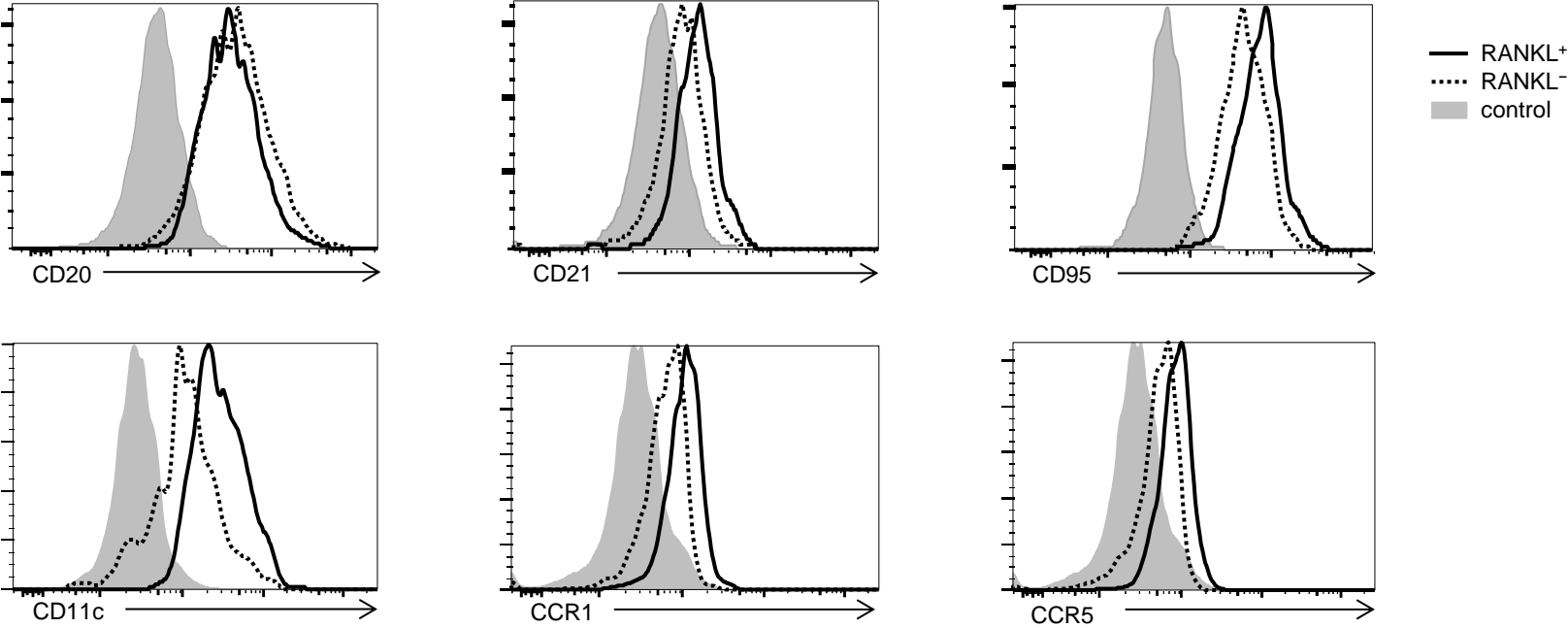

Supplement: Additional file 3: Figure S2. — Phenotypic analysis of RANKL+ and RANKL− effector memory B cells. Purified switched-memory B cells from HC were stimulated with BCR/CD40 and IFN-γ for 48 hours. RANKL+ and RANKL− cells were analyzed for expression of CD20, CD21, CD95, CD11c, CCR1 and CCR5 using respective Abs (all from BioLegend). Representative data are shown (n = 3–4). (PDF 177 kb) [file 13075_2016_957_MOESM3_ESM.pdf]
